# Development of a cypovirus protein microcrystal-encapsulated *Bacillus thuringiensis* UV-tolerant and mosquitocidal δ-endotoxin

**DOI:** 10.1242/bio.059363

**Published:** 2022-09-29

**Authors:** Takumi Ibuki, Satoshi Iwasawa, Ai Ai Lian, Ping Ying Lye, Rina Maruta, Shin-ichiro Asano, Eiji Kotani, Hajime Mori

**Affiliations:** ^1^ Department of Applied Biology, Kyoto Institute of Technology, Sakyo-ku, Kyoto 606-8585, Japan; ^2^ Laboratory of Applied Molecular Entomology, Division of Agrobiology, Graduate School of Agriculture, Hokkaido University, Sapporo 060-8589, Japan

**Keywords:** δ-endotoxin, Polyhedra, Cypovirus, Baculovirus expression system, *Bacillus thuringiensis*

## Abstract

The δ-endotoxin Cry4Aa from *Bacillus thuringiensis israelensis* (*Bti*) has insecticidal characteristics specific to insects of the order Diptera. Although Cry4Aa has shown potential as an effective proteinaceous pesticide against mosquitoes, it has an ultraviolet (UV)-intolerant property that limits its outdoor use. Our previous research showed that protein microcrystal polyhedra from *Bombyx mori* cypovirus can encapsulate diverse foreign proteins and maintain long-term protein activity under hostile environmental conditions, including UV irradiation. In this study, we report the development of polyhedra encapsulating the Cry4Aa insecticidal activity domain by using a modified baculovirus expression system. We confirmed the oral intake of recombinant polyhedra introduced into the experimental environment by the larvae of a mosquito, *Aedes albopictus*, and delivery of encapsulated proteins into the digestive tract. The polyhedra encapsulating partial Cry4Aa showed mosquito larvicidal activity during incubation of larvae with 50% lethal-dose value of 23.717×10^4^ cubes for 10 *Aedes albopictus* larvae in 1 ml water. In addition, polyhedra showed a specific property to reduce the impact of UV-C irradiation on the activity of encapsulated partial Cry4Aa, thus demonstrating the effectiveness of encapsulating *Bti* δ-endotoxins inside polyhedra to increase the availability of proteinaceous pesticides for outdoor use for mosquito control.

## Teaser

**Summary:** *Bacillus thuringiensis israelensis* delta-endotoxin activity domain-encapsulated polyhedra could be a proteinaceous pesticide applicable for mosquito control under outdoor conditions.

## INTRODUCTION

The bacterium *Bacillus thuringiensis* (*Bt*) have the crystal proteins (*Bt* δ-endotoxins), which are composed of three characteristic N-terminal domains that are toxic to insects and the C-terminal domain responsible for their crystallization (Ben-Dov, 2014; Vetter et al., 1998). The crystals have properties to become protoxins by dissolving in the alkaline digestive fluids of many insect larvae, being processed into active toxins by specific proteolysis by the insect digestive enzymes and damaging the midgut columnar cells by mediating their binding to specific receptors (Xu et al., 2014). The detailed molecular mechanisms of the insecticidal activity of δ-endotoxins have been controversial for many years, mainly concerning either hypothesis on midgut programmed cell death (Zhang et al., 2006), or colloid-osmotic lysis (Bravo et al., 2011; Soberón et al., 2007). Since *Bt* spores or *Bt* endotoxins dispersed in agricultural fields were shown to be effective in preventing insect feeding damage to farm products early in the 1950s, they have been used as environmentally conscious bioinsecticides with their insect-specific, highly effective activity (Fisher and Rosner, 1959; Yamamoto, 2001).

Research has shown that irradiation of ultraviolet (UV) in sunlight causes loss of insecticidal activities of δ-endotoxins in the outdoor environment due to UV-induced disruption of indole rings of tryptophan in the δ-endotoxins (Pozsgay et al., 1987; Pusztai et al., 1991). It is considered that UV-induced conformational changes of δ-endotoxins affect association of δ-endotoxins with receptors on the columnar cell membrane. To date, several tests have been conducted for the prevention of UV-induced loss of δ-endotoxin insecticidal activities, using prototypes such as crystals wrapped with oil droplets (Cheung and Hammock, 1985), coated with gelated starches (Dunkle and Shasha, 1989), melanized crystals (Sansinenea and Ortiz, 2015), or mixed with oxidized iron (Asano and Miyamoto, 2007). To develop more practical methods, it is necessary to prolong the longevity of δ-endotoxins dispersed in open and hostile conditions.

*Bombyx mori* cypovirus (BmCPV), a member of the family *Reoviridae*, produces proteinaceous occlusion bodies termed polyhedra, which include microcrystals of the protein polyhedrin with the ability to encapsulate and protect proteins (Ijiri et al., 2009; Ikeda et al., 2001, 2006; Rohrmann, 1986). Progeny viruses are occluded in polyhedra that protect infectivity over the long term, even under harsh environmental conditions in which UVs are irradiated (Coulibaly et al., 2007; Kotani and Mori, 2018). We have previously shown that polyhedra can encapsulate diverse foreign proteins, such as fluorescent proteins (Ijiri et al., 2009), cytokines (Kotani et al., 2015; Maruta et al., 2020; Matsumoto et al., 2014; Matsuzaki et al., 2019; Mori et al., 2007; Nishishita et al., 2011), and fusion proteins comprising enzymes (Ijiri et al., 2010) with an N-terminal polyhedron-targeting signal alpha-helix sequence H1 and C-terminal VP3-tag, which are expressed during polyhedron crystallization in cultured insect cells. The long-term stability of cytokine activity in the polyhedra has been confirmed (Kotani et al., 2015; Matsumoto et al., 2014).

To investigate the effectiveness of the BmCPV polyhedra in protecting the insecticidal activity, H1-tagged δ-endotoxins and BmCPV polyhedra were co-expressed in cultured insect cells to produce polyhedra encapsulating δ-endotoxins. In the present study, we focused on the crystal proteins Cry4Aa from the *Bt* serover *israelensis* (*Bti*), which has insecticidal characteristics specific to insect of the order Diptera such as mosquitoes and flies (Ben-Dov, 2014; Delecluse et al., 1993; Hayakawa et al., 2010; Sen et al., 1988). The control of human infectious diseases such as malaria, dengue fever, and lymphatic filariasis transmitted by mosquito vectors is a global issue that may be solved using specific pesticides to control the population of mosquitoes. This study aimed to investigate whether proteinaceous microcrystal-encapsulated δ-endotoxin activities are preserved by UV irradiation and to develop UV-tolerant effective proteinaceous mosquitocides.

## RESULTS

### Preparation of the recombinant Cry4Aa crystals and Cry4Aa activity domain-encapsulated BmCPV polyhedra

**Fig. 1.**
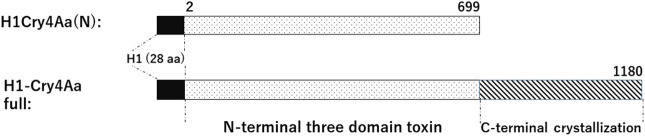
**Schematic representation of the gene for expressed H1-Cry4Aa(N) and H1-Cry4Aa full.** Boxes in black indicate the fused 28 amino acids (aa) of polyhedron-targeting signal H1 alpha-helix sequence from the BmCPV polyhedrin N-terminus. Dotted boxes indicate the N-terminal protoxin domain and three-domain toxic region of Cry4Aa. Dashed boxes indicate C-terminal domain for its crystallization of Cry4Aa.

**Fig. 2.**
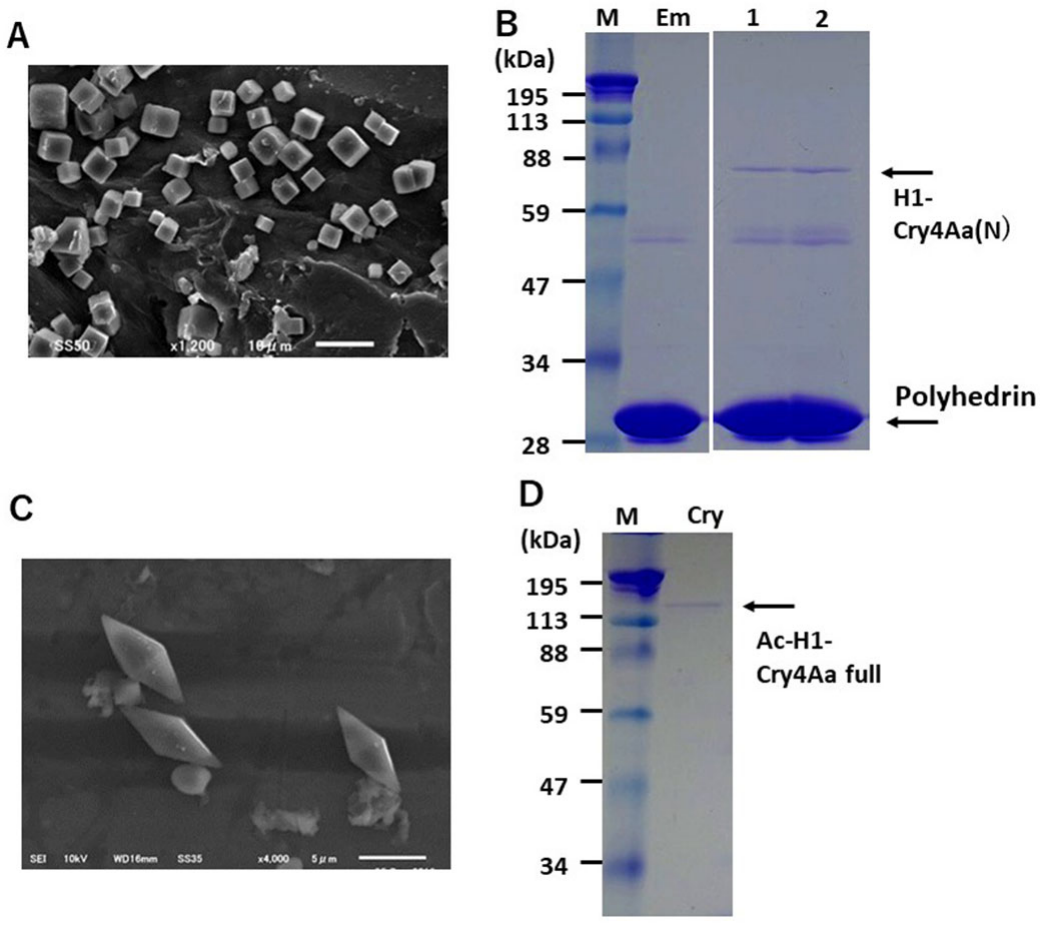
**Expressed H1-Cry4Aa- and H1-Cry4Aa(N)-encapsulated BmCPV polyhedra.** (A) SEM of the isolated Ac-H1-Cry4Aa(N)/polyhedra. Scale bar: 5 µm. (B) SDS-PAGE of the isolated polyhedra. The empty polyhedra as negative control (lane Em), Ac-H1-Cry4Aa(N)/polyhedra (lane 1) and Hy-H1-Cry4Aa(N)/polyhedra (lane 2) prepared in Sf21 cells were separated in a 12.5% gel with the protein size markers (lane M). The arrows indicate H1-Cry4Aa(N)/proteins and polyhedrin. (C) SEM of the isolated H1-Cry4Aa full/crystals. Scale bar: 5 µm. (D) SDS-PAGE of expressed H1-Cry4Aa full/crystals isolated from Sf21 cells (lane Cry) with protein size marker (lane M). The arrow indicates the band for H1-Cry4Aa full. The protein size markers are presented in the left margin.

Cry4Aa from *Bti* is a crystal insecticidal protein specific to insects of the order Diptera such as mosquitoes and flies, which possesses an N-terminal three-domain toxic region and a C-terminal domain responsible for its crystallization (Fig. 1; Delecluse et al., 1993; Hayakawa et al., 2010; Sen et al., 1988). To confer Cry4Aa mosquito larvicidal properties with the UV-resistant characteristics, we produced Cry4Aa N-terminal insecticidal domain (Cry4Aa[N])-encapsulated BmCPV polyhedra that protect protein structure and activity from hostile environmental factors. For this purpose, a fusion protein H1-Cry4Aa(N)/proteins (Fig. 1) of the Cry4Aa(N) and polyhedron-targeting signal alpha-helix sequence H1 were co-expressed with polyhedra (the H29S mutant, as described by Matsuzaki et al., 2019) in IPLB-SF21-AE (Sf21) cells infected with two individual recombinant *Autographa californica* nucleopolyhedrovirus (AcNPV), Ac-H1-Cry4Aa(N)/virus and Ac-CPH29S/virus (construction of all the recombinant baculoviruses is described in the Materials and Methods). Then, the cuboidal polyhedra, Ac-H1-Cry4Aa(N)/polyhedra shown by SEM (Fig. 2A) were isolated.

By recombination of the hybrid-NPV (HyNPV), we also obtained the Hy-H1-Cry4Aa(N)/polH29S/virus that has a property to express both H29S polyhedrin and H1-Cry4Aa(N)/proteins. The Hy-H1-Cry4Aa(N)/polyhedra were produced in Sf21 cells infected with the single Hy-H1-Cry4Aa(N)/polH29S/virus. Sodium dodecyl sulfate-polyacrylamide gel electrophoresis (SDS-PAGE) detected the H1-Cry4Aa(N)/proteins band with an estimated molecular mass of approximately 85 kDa, along with the 32 kDa polyhedrin bands (also observed in the lane Em for the empty polyhedra), which confirmed the encapsulation of polyhedra with H1-Cry4Aa(N)/proteins (Fig. 2B, lane 1). Higher amounts of H1-Cry4Aa(N)/proteins were encapsulated in polyhedra co-expressed in Sf21 cells infected with the single Hy-H1-Cry4Aa(N)/polH29S/virus with knockout of cathepsin and chitinase (Fig. 2B, lane 2) than in cells double-infected with Ac-H1-Cry4Aa(N)/virus and Ac-CPH29S/virus. This result indicates the superiority of using a modified virus with a deficiency of certain protein-degrading enzymes, co-expressing polyhedra, and encapsulated foreign proteins.

In addition, H1-tagged full-length Cry4Aa (Fig. 1) for production of *Bti* crystals, H1-Cry4Aa full/crystals were shown to have the characteristic bipyramid shape under SEM observation (Fig. 2C). The protein band with an estimated molecular mass of approximately 140 kDa (Fig. 2D) was detected by SDS-PAGE analysis of the isolated H1-Cry4Aa full/crystals (Fig. 2C), confirming the crystal formation of the recombinant proteins expressed from the baculovirus vector in Sf21 cells.

### Oral intake of the recombinant polyhedra by *Aedes albopictus* larva

**Fig. 3.**
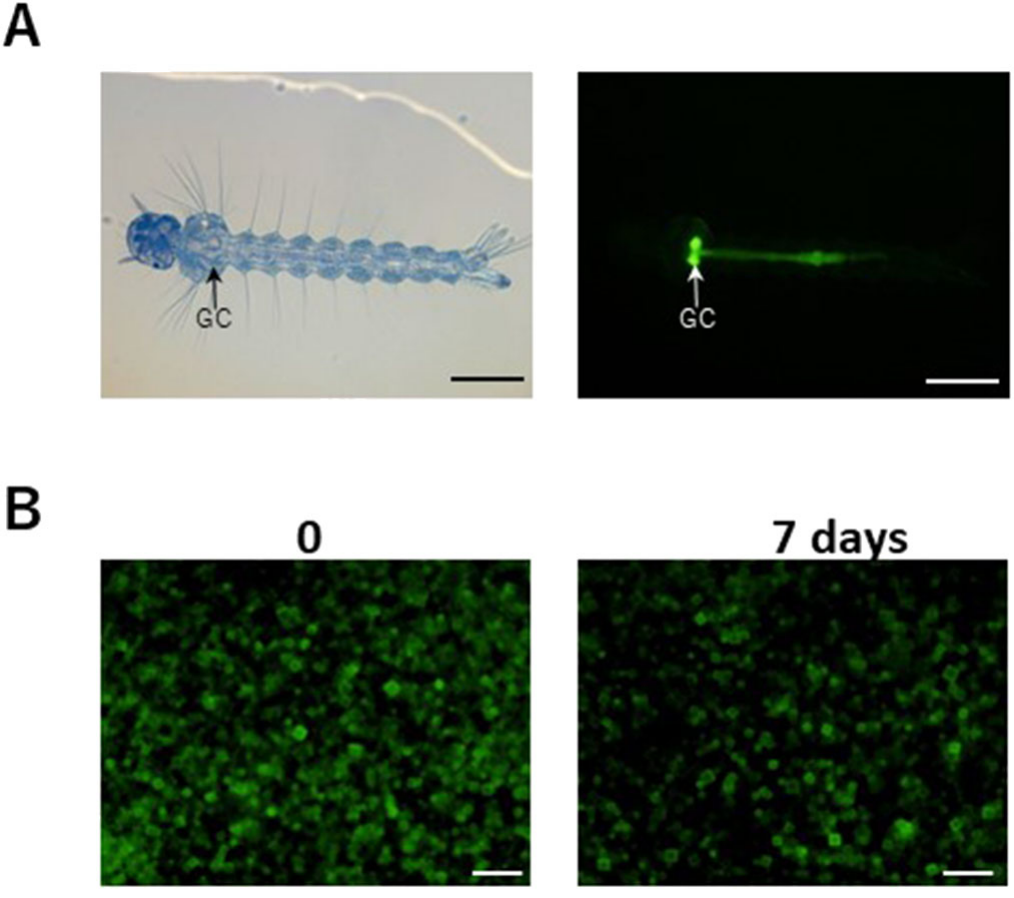
**Oral intake of the recombinant H1-EGFP/polyhedra by *A. albopictus* larva.** (A) Light (left) and fluorescent microscopic observation (right) of second-instar day 1 *Aedes albopictus* larva kept with recombinant H1-EGFP/polyhedra for 1 d (10×objective lens). GC: gastric cecum in thorax. Scale bar: 1000 µm. (B) Fluorescence microscopy (40×objective lens) of the H1-EGFP/polyhedra introduced into the environment keeping *A. albopictus* larva in a well after 0 and 7 d incubation. Scale bar: 30 µm.

To investigate the oral intake of recombinant polyhedra by mosquitoes, prepared H1-EGFP-encapsulated polyhedra (H1-EGFP/polyhedra; 1×10^5^ cubes·200 µl^−1^) were added to a 96-well plate containing two of day 1 second instar *A. albopictus* larvae. On day 2 of the second instar, strong EGFP fluorescence was observed in the gastric cecum in the thorax digestive tract of the larva, and the weaker EGFP fluorescence in the midgut in the abdomen (Fig. 3A; see Boudko et al., 2001 for inner morphology of the closely relative mosquito, *A. aegypti* larva). EGFP was gradually degraded from the anterior (first abdominal segment) to the central midgut (fifth abdominal segment) and almost disappeared in the posterior midgut, indicating that the polyhedra were orally taken into the *A. albopictus* larva, and EGFP released from the degraded polyhedra was gradually digested inside the midgut.

Next, we prepared 3×10^5^ cubes of H1-EGFP/polyhedra, and introduced them to a 96-well plate where an *A. albopictus* larva was maintained for 7 d from the second instar day 1. Observation of the basement of the well showed that the introduced polyhedra clearly decreased after a mosquito larva was maintained for 7 d (Fig. 3B). The reduction rate of the EGFP intensity after 7 d was shown to be approximately 44.60% (*n*=3), as determined by the ImageJ analysis.

### Mosquito larvicidal activity of the recombinant polyhedra

**Fig. 4.**
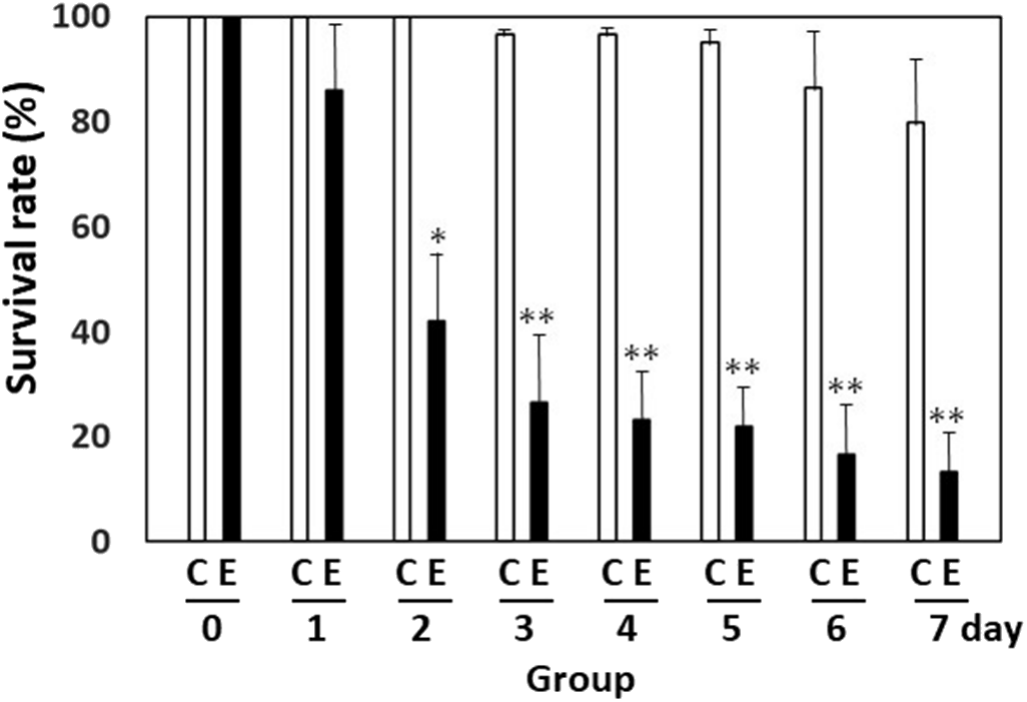
**Rate of the survived *Aedes albopictus* larvae in the assay for the mosquito larvicidal activity of the Ac-H1-Cry4Aa(N)/polyhedra (group E) produced in the Sf21.** Ten second-instar *A. albopictus* larvae in 1 ml water were kept with 1.5×10^6^ cubes of Ac-H1-Cry4Aa(N)/polyhedra produced in Sf21 cells. The same number of empty polyhedra were used as controls (group C). Survival rates (%) of the larvae were measured. Data shown are the means±s.d. (*n*=3 independent samples). *P<0.005 and **P<0.001 versus the control group on each day.

To investigate the mosquito larvicidal activity of recombinant polyhedra, the effects of Ac-H1-Cry4Aa(N)/polyhedra (1.5×10^6^ cubes) on ten second-instar *A. albopictus* larvae kept in 1 ml water were compared with those receiving the same number in the control of empty polyhedra for 7 d. Experiments in each group were performed thrice for statistical analysis. Just after the 7 d incubation, all the survivors in the control group started to pupate. However, a few individuals failed ecdysis and died in the control group, probably because of the impracticability of the environment with the normal development of *A. albopictus*. A higher lethal effect of the Ac-H1-Cry4Aa(N)/polyhedra-treated group was detected on each day, compared to the control empty polyhedra-treated groups (Fig. 4). From day 3 to day 7 in the assay period, approximately 56.67 to 66.67% of the lethality of Ac-H1-Cry4Aa(N)/polyhedra treated group was detected, compared with that of empty polyhedra treated group (*P<*0.001, *n*=3; Fig. 4), indicating the effectiveness of Ac-H1-Cry4Aa(N)/polyhedra larvicidal activity on the *A. albopictus* larvae.

Next, we investigated the LD50 of Ac-H1-Cry4Aa(N)/polyhedra and Hy-H1-Cry4Aa(N)/polyhedra produced in Sf21 using a Probit method (Finney, 1971) measuring effects of the increasing number of polyhedra on ten *A. albopictus* larvae in 1 ml water. Table 1 highlights the LD50 value for each group with confidence limits. Higher lethality of Hy-H1-Cry4Aa(N)/polyhedra compared with Ac-H1-Cry4Aa(N)/polyhedra was observed, reflecting the difference in the quantity of encapsulated proteins presented in Fig. 2C.

**Table 1.**
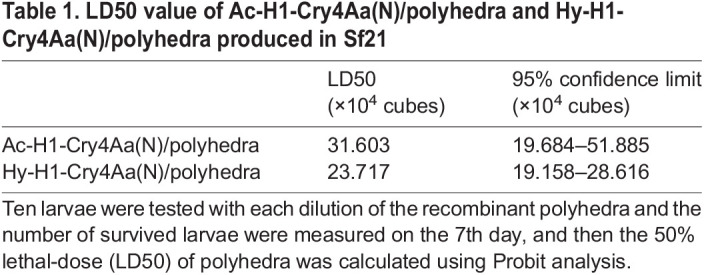
LD50 value of Ac-H1-Cry4Aa(N)/polyhedra and Hy-H1-Cry4Aa(N)/polyhedra produced in Sf21

### Impact of UV-C irradiation to recombinant polyhedra on the mosquito larvicidal activity

**Fig. 5.**
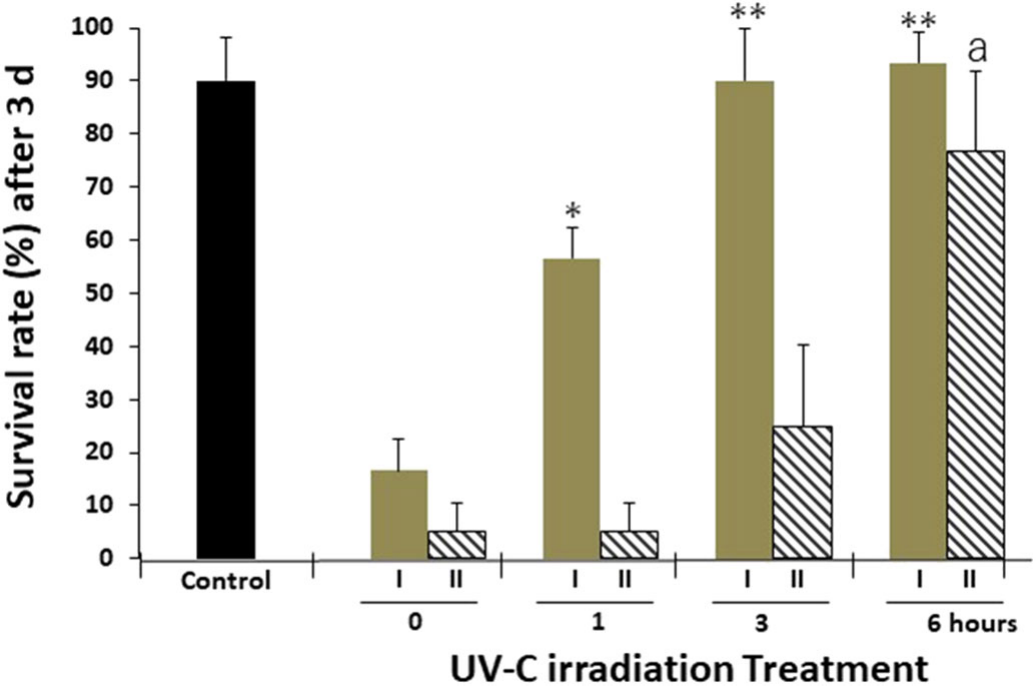
**Effects on the larvicidal activity of recombinant proteins by UV-C irradiation.** H1-Cry4Aa full/crystals (5×10^6^ crystals; column I) or Hy-H1-Cry4Aa(N)/polyhedra (5×10^6^ cubes; column II), irradiated by UV-C for 0, 1, 3, and 6 h, were air-dried on the bottom of the well and ten *A. albopictus* larvae were incubated in 1 ml water in the well. Larvae with no recombinant proteins in 1 ml water were used as a control. Survival rate (%) of the larvae were measured after 3 d incubation. Data shown are the means±s.d. (*n*=3 independent samples). *P<0.01 and **P<0.001 versus 0 h treated-H1-Cry4Aa full/crystals; ^a^*P*<0.001 versus 0 h treated-Hy-H1-Cry4Aa(N)/polyhedra.

To determine whether UV irradiation affects the mosquito larvicidal activities of the H1-Cry4Aa full/crystals or Hy-H1-Cry4Aa(N)/polyhedra, these proteins were placed in a 24-well plate, air-dried, irradiated with UV-C light, and then introduced into the three-round bioassays measuring their larvicidal activity for 3 d against ten second-instar *A. albopictus* larvae kept in 1 ml water in 24-well plates. Approximately 10% of larvae were shown to die in the control group with no recombinant proteins in the condition. UV-C irradiation of Ac-H1-Cry4Aa full/crystals for 1, 3, and 6 h showed 36.67% (*P*<0.01), 70.00% (*P*<0.001), and 73.34% (*P*<0.001) reductions in larvicidal activity, respectively (Fig. 5; *n*=3). On the other hand, UV-C-irradiation of Hy-H1-Cry4Aa(N)/polyhedra for 1, 3, and 6 h showed 0.00%, 16.67%, and 70.00% (*P<*0.001) reductions in larvicidal activity, respectively (Fig. 5). Higher protection effects on H1-Cry4Aa(N)/proteins larvicidal activity against 1 and 3 h UV-C irradiation by encapsulation in polyhedra were shown compared with H1-Cry4Aa full/crystals, indicating the strong effect of polyhedra in protecting the larvicidal activity of Cry4Aa(N) protein from UV-C irradiation.

## DISCUSSION

In the present study, we developed Cry4Aa N-terminal toxic domain-encapsulated BmCPV polyhedra produced in cultured Sf21 insect cells (Fig. 2). Recombinant polyhedra were introduced into the experimental environment, using *A. albopictus* second-instar larvae for bioassay of larvicidal activities. It is still unclear how the addition of the H1-tag to the N-terminus of Cry4Aa(N) affects its bioactivity to kill *A. albopictus* larvae, but thus far we have not found a remarkable activity loss of some encapsulated proteins in several kinds of recombinant polyhedra, including cytokines and enzymes (Kotani et al., 2015; Maruta et al., 2020; Matsumoto et al., 2014; Matsuzaki et al., 2019; Mori et al., 2007; Nishishita et al., 2011).

Although the H1-EGFP/polyhedra sunk to the bottom of the plate, where larval contact with polyhedra was not frequent, the number of H1-EGFP/polyhedra was reduced by larval oral intake (Fig. 3). In addition, strong EGFP fluorescence was observed in the thorax gastric cecum, weak fluorescence in the abdominal central midgut, and gradual degradation of the fluorescence in the posterior midgut (Fig. 3). Previously, *in situ* analysis of pH gradients in *A. aegypti* using pH-sensitive microelectrodes revealed alkalescent conditions inside the mosquito thorax gastric cecum at around pH 8 and strong alkaline conditions inside the midgut at around pH 10 (Boudko et al., 2001). The BmCPV polyhedra with mutation H29S are least soluble in the solution at pH 8, and are well soluble in pH 10 aqueous fluid where polyhedra spontaneously disassemble to release encapsulated proteins to the outside. This evidence is consistent with the result in Fig. 3A that EGFP fluorescence remained stable in the thorax and disappeared in the midgut where polyhedra could not protect the encapsulated protein against digestion.

Similarly, H1-Cry4Aa(N)-encapsulated polyhedra were found to protect the larvicidal activity of encapsulated proteins and migrate to the midgut, such that larvicidal activities were observed on *A. albopictus* (Fig. 4). These results indicate that efficient release of H1-Cry4Aa(N)/proteins from polyhedra could locally occur in the midgut digestive juice, and active H1-Cry4Aa(N)/proteins could damage the midgut epithelial columnar cells (Xu et al., 2014), even though the molecular mechanisms by which *Bt* δ-endotoxins damage midgut cells are still unclear (Bravo et al., 2011; Zhang et al., 2006). However, *Bti* δ-endotoxins such as Cry4Aa or Cry11A have mosquito-specific activities compatible with killing *Aedes* larvae that mediate dengue fever and malaria, which depend on their possible molecular characteristics to specifically interact with the mosquito midgut epithelial cells (Ben-Dov, 2014). Baculovirus virions do not appear to be present inside polyhedra because they have no sequential characteristics for their encapsulation (Kotani and Mori, personal communication, 2022). Therefore, there would be an advantage that the polyhedra are biological pesticides that do not transmit baculovirus to the environment and expand the threats of the infectious disease for lepidopteran insects. Polyhedra themselves are biologically harmless, as Figs 4 and 5 show, empty polyhedra were not infective, without remarkably influencing the developmental process of *A. albopictus* larvae, similar to the untreated control. Thus, H1-Cry4Aa(N)-encapsulated polyhedra are ecologically conscious and mosquito specific. Additionally, it is suggested that the use of polyhedra technology will be applicable in the construction of delivery systems accompanied by the shifting of pH conditions in the body of not only living insects, but also widely diverse animal kinds on artificial regulation of the biological mechanisms from outside.

Next, by analyzing the impact of UV irradiation on H1-Cry4Aa(N)-encapsulated polyhedra, polyhedra were shown to protect the larvicidal activity of H1-Cry4Aa(N) against UV irradiation (Fig. 5). The larvicidal activity of H1-Cry4Aa full/crystals prepared from recombinant baculovirus-infected Sf21 cells was clearly reduced by UV-C irradiation treatment. UV-C irradiation for 3 h under the same conditions caused complete inhibition of the activity of commercial *Bti* mosquitocide crystals (20 µg/well of Mosquito dunks; Summit Chemical, Baltimore, MD, USA; data not shown), indicating the disadvantageous properties that crystallization domain of *Bti* δ-endotoxin could not protect the inside insecticidal activities against UV irradiation. Previously, Ijiri et al. (2009) detected fluorescent proteins with N-terminal H1 in baculovirus-produced polyhedra, demonstrating that H1-fused foreign proteins were encapsulated inside the polyhedra owing to the H1 property associated with the polyhedrin groove domain consisting of the tyrosine cluster to tightly link the polyhedrin to each other during polyhedron crystallization. Polyhedra can protect substances, including BmCPV virions, under harsh conditions where UVs are irradiated (Rohrmann, 1986). Structural analysis of Cry4Aa has suggested that the domain I-domain II interface region, which is important for the interaction of cell receptors, consists of surface hydrophobic patches including many aromatic amino acids like tryptophan (Boonserm et al., 2006). UV irradiation-induced disruption of the indole ring of tryptophan is closely related to the loss of insecticidal activities of δ-endotoxins (Pozsgay et al., 1987; Pusztai et al., 1991). Based on this evidence, it is likely that the predicted receptor binding region of Cry4Aa to interact with the mosquito columnar cell receptors needs to have the structural basis of UV sensitivity to negatively impact larvicidal activity. Our results also suggest that the encapsulation of polyhedra in the Cry4Aa larvicidal domain is more effective in protecting these important structures than in its own original crystal. To date, several chemical modifications of *Bt* δ-endotoxins have been performed to prevent UV-induced activity loss (Dunkle and Shasha, 1989; Asano and Miyamoto, 2007; Sansinenea and Ortiz, 2015). However, effective methods to improve UV tolerance are still elusive. *Bt* toxins synthesized in transgenic plants are thought to be highly resistant to UV irradiation, indicating that this technology has become the best solution to this problem (Soares and Quick, 1990). Accordingly, many genetically modified crop plants with the ability to produce *Bt* toxins in their leaf cells have been established. However, in the case of mosquitoes, larvae are not herbivorous.

In addition, there will still be important investigations of another *Bti* toxin, such as Cry11A, without possession of the C-terminal crystallization domain (Ben-Dov, 2014), focusing on the encapsulation of Cry11A inside polyhedra to preserve its larvicidal activity from UV-induced inactivation. In addition, a test to evaluate the larvicidal activity of polyhedra encapsulating *Bti* δ-endotoxins needs to be performed on several mosquito types that mediate mammalian viral diseases. However, the insight gained from the present study would become a basis for developing an effective UV-tolerant mosquitocide protein for outdoor use in the future. Thus, this evidence demonstrates that *Bti* δ-endotoxin activity domain-encapsulated polyhedra could be a proteinaceous pesticide applicable for mosquito control under outdoor conditions.

Additionally, the quantitative extent of polyhedra encapsulating crystal δ-endotoxins is still under consideration for practical use. Modified HyNPV has the ability to infect silkworms, including pupae, a competent baculovirus host. For the industrial mass production of recombinant protein production systems, silkworm pupae have often been used as infectious hosts because of the utility of pupae with soft epithelium for the effective isolation of recombinant proteins and with higher protein production efficiency than the use of cultured cells. In a previous study, we generated transgenic silkworms with deficient posterior silk glands, which could not synthesize the silk protein fibroin and had a genetic trait with heavier pupal weight storing the nutrition for fibroin synthesis (Otsuki et al., 2017). Heavier pupae are expected to be hosts for more effective foreign protein production. In addition, recent advances in polyhedra technology have focused on the effective preparation of cytokine-encapsulated polyhedra from the silk glands of transgenic silkworms without a remarkable loss of their biological activity (Kotani et al., 2015; Honda et al., 2020). The large-scale production of recombinant *Bti* toxin-encapsulated polyhedra will be practical by using a specific silkworm pupa as a baculovirus host, with the established sericultural facility to provide sufficient silkworm pupae and contribute for giving a clue to the development of superior proteinaceous mosquitocides that are useful worldwide.

## MATERIALS AND METHODS

### Cells and insects

To express the BmCPV polyhedra-encapsulated Cry4Aa sequence (Sen et al., 1988; Acc. number BAA00179), we used *Spodoptera frugiperda* ovary-derived culture cells named IPLB-SF21-AE cells (Sf21). Sf21 cells were cultured in the TC-100 medium supplemented with 10% fetal bovine serum at 27°C.

Larvae from fertilized eggs of the mosquito *Aedes albopictus* (Sumika Technoservice, Hyogo, Japan) were reared in water containing inactivated dry yeast powder (1 mg ml^−1^). For the larvicidal activity of recombinant protein crystals assay, each emerged larva at second instar was kept in 1 ml of water in 96-well or 24-well plates, with inactivated dry yeast powder (0.5 mg ml^−1^) at 27°C, under the photoperiodic conditions, 16L:8D. The water with inactivated dry yeast powder in each well was changed daily. For incubation of larvae in all the bioassays of this work, we used the water containing the same concentration of inactivated dry yeast.

### Construction of transfer vectors and preparation of the recombinant baculovirus

The oligonucleotide primers used in this study are listed in Table 2. A Cry4Aa cDNA clone was used (Sen et al., 1988). To construct a donor plasmid in the Gateway Technology System (Invitrogen, Carlsbad, CA, USA), a DNA fragment encoding full-length Cry4Aa amino acid sequence (Fig. 1) was amplified by PCR, using the primers attB1 Cry4Aa (forward) and attB2 Cry4Aa full (reverse), and cloned into the plasmid pDONR221 by a BP-reaction of the Gateway system to construct a pDONR-Cry4Aa full. A DNA fragment encoding the N-terminal three-domain toxic region lacking a domain for its crystallization [Cry4Aa(N); Fig. 1] was amplified by PCR with the primers attB1 Cry4Aa (forward) and attB2 Cry4Aa(N) (reverse) and similarly cloned into pDONR221 to obtain pDONR-Cry4Aa(N). The inserted sequence between the attB1 to attB2 sequences in both plasmids was cloned into pDEST(N)H1 (Ijiri et al., 2009) by LR reaction of the Gateway system to obtain the baculovirus transfer vectors pTrans-H1-Cry4Aa full and pTrans-H1-Cry4Aa(N). Both transfer vectors were designed to encode the N-terminal polyhedron-encapsulating signal H1 sequence (Coulibaly et al., 2007; Ijiri et al., 2009) and Cry4Aa-derived sequence in the frame downstream of the NPV polyhedrin promoter. The transfer vector pTrans-H1-Cry4Aa full or pTrans-H1-Cry4Aa(N) was co-transfected into Sf21 cells with the BaculoGold^TM^ linearized baculovirus DNA (BD Biosciences, San Jose, CA, USA). Recombinant baculovirus, Ac-H1-Cry4Aa full/virus, and Ac-H1-Cry4Aa(N)/virus were obtained for NPV polyhedrin promoter driven-expression of full-length H1-Cry4Aa full/proteins and H1-Cry4Aa(N)/proteins by the three-round plaque purification, multiplied by infection of Sf21 cells, harvested, and stored at 4°C.

**Table 2.**
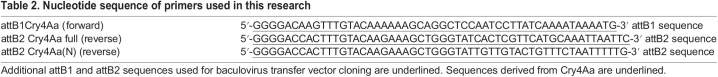
Nucleotide sequence of primers used in this research

The plasmid pTrans-H1-Cry4Aa(N) was also co-transfected into Sf21 cells with the *Bsu36I*-treated linearized DNA from modified hybrid baculovirus, designated as modified HyNPV (Mori et al., 1992), which was provided by Protein Crystal (Kyoto, Japan). It has the ability to infect both Sf21 and *B. mori* cells, and carries disrupted chitinase and cathepsin genes by replacement with a sequence for expression of BmCPV H29S modified polyhedrin with an amino acid mutation (H29S) under the control of the NPV polyhedrin promoter. The linearized modified HyNPV also carried truncated ORF1629 for the virion protein necessary for replication, so that only a recombinant virus caused by the homologous recombination of co-transfected transfer vectors for recovery of ORF1629 could acquire the ability to replicate in insect cells. Then, the Hy-H1-Cry4Aa(N)/polH29S/virus was obtained by co-transfection of the modified HyNPV DNA and transfer vector followed by three rounds of plaque purification methods. The Hy-H1-Cry4Aa(N)/polH29S/virus has a property to co-express both H29S polyhedrin and H1-Cry4Aa(N) proteins.

### Expression of Cry4Aa protein-encapsulating BmCPV polyhedra in insect cells

The recombinant *Autographa californica* NPV (AcNPV) designed to express the BmCPV H29S polyhedrin under control of the polyhedrin promoter [Ac-CPH29S/virus; multiplicity of infection (m.o.i)=5; Matsuzaki et al., 2019] was co-infected into Sf21 cells (2×10^8^) with the Ac-H1-Cry4Aa(N)/virus (m.o.i=5) to obtain the Ac-H1-Cry4Aa(N)-encapsulated polyhedra (Ac-H1-Cry4Aa[N]/polyhedra). For production of only H29S polyhedra (empty polyhedra), Sf21 cells were infected with single Ac-CPH29S/virus. Sf21 cells (2×10^8^) were also infected with the Ac-H1-Cry4Aa full/virus for preparation of the H1-Cry4Aa full/crystals. Recombinant virus expressing EGFP-fused with N-terminal H1 was co-infected into Sf21 with the AcCPH29S/virus to produce H1-EGFP encapsulated-polyhedra (H1-EGFP/polyhedra) using the method described by Ijiri et al. (2009). In addition, Hy-H1-Cry4Aa(N)/polH29S/virus (m.o.i=10) was similarly infected into Sf21 cells to obtain Hy-H1-Cry4Aa(N)/polyhedra. Cells infected with these recombinant viruses were suspended in the culture medium, harvested, and centrifuged at 2300×***g*** for 5 min. The supernatant was discarded, and then cells were re-suspended in a 15 ml phosphate buffered-saline (PBS, pH 7.2) and repeatedly treated with a VP 050N ultrasonic homogenizer (TAITEC, Aichi, Japan) at 6% power for 30 s. The recombinant polyhedra and H1-Cry4Aa full/crystals were separated by centrifugation and the supernatant of the cell homogenate was removed. The pellet was dissolved in PBS and further treated with an ultrasonic homogenizer until the purification of recombinant polyhedra and H1-Cry4Aa full/crystals was completed.

For scanning electron microscopy (SEM), Ac-H1-Cry4Aa(N)/polyhedra or H1-Cry4Aa full/crystals were placed on an aluminum plate and treated by gold sputtering. Images were obtained via SEM (200 Å) using a JSM-6510 (JEOL, Tokyo, Japan).

For SDS-PAGE analysis, 2×10^4^ prepared empty polyhedra, Ac-H1-Cry4Aa(N)/polyhedra, Hy-H1-Cry4Aa(N)/polyhedra, or 2×10^2^ H1-Cry4Aa full/crystals were suspended in 50 µl of SDS-PAGE sample buffer, boiled for 5 min, and subsequently chilled on ice. Each sample (20 µl) was separated by electrophoresis on a 12.5% SDS-PAGE gel and detected using Coomassie Brilliant Blue staining.

### Bioassay of recombinant protein crystals

Prepared H1-EGFP/polyhedra (1×10^5^ cubes 200 µl^−1^) were added to a 96-well plate containing two day 1 second-instar *A. albopictus* larvae. After incubation at 27°C for 24 h, the larvae were placed on a glass slide, and the EGFP fluorescence in the body was detected by irradiation with fluorescent light at a wave length of 490 nm under a fluorescence microscope with Olympus IX71 (Olympus Corporation, Tokyo, Japan) with a 10× objective lens.

A second-instar day 1 *A. albopictus* larva was placed in a well of a 96-well plate with a flat bottom, at which 3×10^5^ H1-EGFP/polyhedra were absorbed, recombinant polyhedra remaining at the bottom of the well after 7 d incubation were detected under a fluorescence microscope with a 40×objective lens. The reduction of the EGFP fluorescence of the observation was investigated by using the computer assisted image analysis by using the ImageJ software (NIH, Bethesda, MD, USA; http://imagej.nih,gov/ji/).

Ten second-instar *A. albopictus* larvae in 1 ml of water were placed in a 24-well plate with Ac-H1-Cry4Aa(N)/polyhedra or Hy-H1-Cry4Aa(N)/polyhedra, and the effects of recombinant polyhedra on larval survival rate (%) were compared with that of the same number of control empty polyhedra produced in Ac-CPH29S/virus infected-Sf21. Ten larvae were tested with each dilution of the recombinant polyhedra, survival rate was examined on 7th day, and then the 50% lethal-dose (LD50) of polyhedra was calculated by the Probit analysis (Finney, 1971). Cannibalistic behavior of larvae to other living individuals was not observed in this condition.

### Effects of ultraviolet (UV-C) light on the mosquito activity of recombinant polyhedra

Recombinant proteins, Hy-H1-Cry4Aa(N)/polyhedra (5×10^6^), and the same number of H1-Cry4Aa full/crystals were placed on the bottom of the 24-well plate and then irradiated with UV-C light emitted from a 20 cm distant 60 W h^−1^ germicidal fluorescent light applied at 100 V for 1 to 6 h. After UV-C irradiation, these protein crystals were used in the mosquitocide assay described below.

### Data statistical analysis

The results in Figs 4 and 5 are expressed as mean±standard deviation (s.d.) of triplicate assays (*n*=3 independent samples). Data were analyzed using one-way analysis of variance followed by Tukey's *post-hoc* test for pairwise comparisons. Differences were considered statistically significant at *P<*0.01.
